# The Role of Extraversion and Psychopathological Symptoms in Drug Resistance in Psychiatric Patients: A Preliminary Study

**DOI:** 10.1192/j.eurpsy.2025.819

**Published:** 2025-08-26

**Authors:** M. N. Modesti, J. F. Arena, A. Del Casale, S. Ferracuti, C. Guariglia, S. De Persis

**Affiliations:** 1 Department of Psychology; 2 Department of Dynamic and Clinical Psychology and Health Studies; 3 Department of Human Neuroscience, Sapienza University of Rome; 4 Cognitive and Motor Rehabilitation and Neuroimaging Unit, “ Santa Lucia” Scientific Institute for Research, Rome; 5 Department for the Protection and Promotion of Mental Health, Local Health Unit, Rieti, Italy

## Abstract

**Introduction:**

Drug resistance is a major clinical challenge in psychiatry, with limited understanding of influencing factors. Personality traits and psychopathological symptoms may contribute to drug resistance, affecting treatment response to standard interventions.

**Objectives:**

This study aims to assess whether personality traits, mainly neuroticism and extraversion, and psychopathological symptoms correlate with drug resistance profiles in clinically stable patients with severe mental disorders.

**Methods:**

The study included 36 outpatients (17 males, 19 females) consecutively treated at the Mental Health Centre of Rieti, Italy. Patients were diagnosed with Major Depressive Disorder (39%), Bipolar Disorder (25%), Schizophrenia (28%), and other diagnoses, including Obsessive-Compulsive Disorder (8%). Drug resistance was defined as a lack of response to previous antidepressant treatments, requiring either dual antidepressant therapy, add-on therapy with a tricyclic or lithium, or a lack of response to atypical antipsychotic treatments, necessitating either dual atypical antipsychotic therapy, the addition of a typical antipsychotic, or the prescription of clozapine. Mann-Whitney U tests were used to compare 11 patients with a drug resistance profile to the remaining 25, and stepwise logistic regression was conducted to assess the association between drug resistance (dependent variable) and study variables, including the Brief Psychiatric Rating Scale (BPRS), Eysenck Personality Questionnaire factors (e.g., extraversion, neuroticism), and Global Assessment of Functioning (GAF) scores. The local ethics committee approved this study (Protocol No. 0948/2023).

**Results:**

Mann-Whitney U tests revealed significant differences between groups in total BPRS scores (p = 0.032) and the BPRS Negative Symptoms subscale (p = 0.001), with higher scores in the drug-resistant group. GAF scores also differed significantly (p = 0.022), with lower scores in resistant patients. Logistic regression showed that extraversion had a significant negative association with drug resistance (β = -0.803, p = 0.033), suggesting higher extraversion is linked to reduced resistance. The BPRS Negative Symptoms factor had a significant positive association (β = 0.467, p = 0.026), while Positive Symptoms showed a trend toward a positive relationship (β = 0.508, p = 0.059). The final model explained a substantial proportion of variance (McFadden’s R² = 0.543) and improved over previous models (ΔΧ², p = 0.042).

**Conclusions:**

Extraversion negatively correlates with drug resistance profiles in clinically stable patients with severe mental disorders. BPRS negative symptoms are positively correlated with resistance, and positive symptoms show a similar trend. This study highlights the importance of personality and psychopathological aspects in treatment response and the need for personalized interventions for patients with drug resistance.

**Disclosure of Interest:**

None Declared

